# Performances of Different Fragment Sizes for Reduced Representation Bisulfite Sequencing in Pigs

**DOI:** 10.1186/s12575-017-0054-5

**Published:** 2017-06-07

**Authors:** Xiao-Long Yuan, Zhe Zhang, Rong-Yang Pan, Ning Gao, Xi Deng, Bin Li, Hao Zhang, Per Torp Sangild, Jia-Qi Li

**Affiliations:** 1 Guangdong Provincial Key Lab of Agro-Animal Genomics and Molecular Breeding, National Engineering Research Centre for Breeding Swine Industry, College of Animal Science, South China Agricultural University, Guangzhou, Guangdong China; 2 Section of Comparative Pediatrics and Nutrition, Department of Veterinary Clinical and Animal Sciences, University of Copenhagen, Frederiksberg, Denmark; 3 Department of Animal Sciences, Georg-August University, Albrecht Thaer-Weg 3, Göttingen, Germany

**Keywords:** DNA methylation, RRBS, Different fragment sizes, Pigs

## Abstract

**Background:**

Reduced representation bisulfite sequencing (RRBS) has been widely used to profile genome-scale DNA methylation in mammalian genomes. However, the applications and technical performances of RRBS with different fragment sizes have not been systematically reported in pigs, which serve as one of the important biomedical models for humans. The aims of this study were to evaluate capacities of RRBS libraries with different fragment sizes to characterize the porcine genome.

**Results:**

We found that the *Msp*I-digested segments between 40 and 220 bp harbored a high distribution peak at 74 bp, which were highly overlapped with the repetitive elements and might reduce the unique mapping alignment. The RRBS library of 110–220 bp fragment size had the highest unique mapping alignment and the lowest multiple alignment. The cost-effectiveness of the 40–110 bp, 110–220 bp and 40–220 bp fragment sizes might decrease when the dataset size was more than 70, 50 and 110 million reads for these three fragment sizes, respectively. Given a 50-million dataset size, the average sequencing depth of the detected CpG sites in the 110–220 bp fragment size appeared to be deeper than in the 40–110 bp and 40–220 bp fragment sizes, and these detected CpG sties differently located in gene- and CpG island-related regions.

**Conclusions:**

In this study, our results demonstrated that selections of fragment sizes could affect the numbers and sequencing depth of detected CpG sites as well as the cost-efficiency. No single solution of RRBS is optimal in all circumstances for investigating genome-scale DNA methylation. This work provides the useful knowledge on designing and executing RRBS for investigating the genome-wide DNA methylation in tissues from pigs.

## Background

In mammals, DNA methylation preferably occurs at CpG dinucleotides, and it modifies many key biological processes, including gene transcription [1], genomic imprinting [2], tissue differentiation [3] and phenotypic variation [4]. By profiling the DNA methylome, it is possible to mirror the epigenetic patterns that may regulate gene expression. However, the asymmetric distribution of CpG sites and the short length of sequencing reads make whole-genome bisulfite sequencing (WGBS) relatively costly [5]. Therefore, reduced representation bisulfite sequencing (RRBS) is developed, which uses *Msp*I, a restriction enzyme that cuts C|CGG sites, to select the CG-rich regions and reduces the required amount of sequencing to study the genome-wide DNA methylation [6, 7].

RRBS has a low cost for per detected CpG site, and it is highly sensitive to low DNA input, while providing single-nucleotide resolution to quantify the DNA methylation level distribution [7, 8]. The cost-effective RRBS processes allow for large-scale mapping of DNA methylation in a large number of samples (e.g. >100 per week) [9]. Recently, RRBS has been performed on samples from humans [9], pigs [10], sheep [5] and many model organisms [11–13] to generate the genome-scale DNA methylation and screen the dynamic changes in the methylomes. Additionally, the DNA methylation information obtained by RRBS could be scaled up to WGBS data [14] and used in studies on methylation quantitative trait loci [15]. New approaches and developments based on RRBS have been developed, such as the laser capture microdissection-RRBS [16], single-cell RRBS [17], double-enzyme RRBS [18], and high-throughput targeted repeat element RRBS [19], to profile the genome-wide DNA methylation of the representative genome or of the specific genomic features. These processes and developments have greatly advanced investigations of DNA methylomes in mammals.

In the vertebrate genome, the fragment size of 40–220 bp has been suggested for RRBS [20]. The applications and technical analyses of RRBS with this fragment size, such as the genomic coverage and coverage depth, have been systematically investigated in mice [20] and humans [21]. Furthermore, performances and technical assessments of RRBS with different fragment sizes but not the 40–220 bp fragment size have also been discussed to resolve the fragment size selection and sequence depth in livestock, e.g. sheep [5]. However, the applications and technical performances of RRBS with different fragment sizes have not been systematically reported in pigs, which serve as one of the important biomedical models for humans [10, 22].

In this study, we attempted to report and discuss technical applications of RRBS with different fragment sizes on the sample from pigs. We first bioinformatically predicted distribution characteristics of the different *Msp*I-digested fragment sizes of 40–110 bp, 110–220 bp and 40–220 bp in the porcine genome, respectively. Then, RRBS libraries were built with these fragment sizes from the same porcine DNA sample, and these RRBS libraries were sequenced to show the mapping efficiencies, optimal sequencing quantities, and distributions of the detected CpG sites across the locations of genes and CpG islands (CGIs) at the genome scale. This work would provide the methodological information about RRBS for use in epigenomic investigations of pigs, and it sheds additional light on how to design RRBS with the appropriate fragment size for comprehensively representing the methylome of pigs.

## Methods

### Ethic Statement

The ovary was collected from one female Landrace × Yorkshire crossed gilt aged 180 days. Pig cares and experiments were approved by the Animal Care and Use Committer of the South China Agricultural University, Guangzhou, China (approval number: SCAU#2013–10). All experiments and conductions were performed in accordance with the guidelines and regulations of the Administration of Affairs Concerning Experimental Animals (Ministry of Science and Technology, China, revised in June 2004).

### Simulation of *Msp*I Digestion and Size Selection

The porcine reference genome (Sscrofa 10.2), which was downloaded from the Ensembl Genome Browser (http://www.ensembl.org/Sus_scrofa/Info/Index), was digested using *Msp*I in the simulation. The restriction enzyme cutting site for *Msp*I was C|CGG. The single sequences between two consecutive restriction sites were extracted as a *Msp*I-digested segment. The fragment size of 40–220 bp was recommended for RRBS in mammalian genomes, and based on this, fragment sizes of 40–110 bp, 110–220 bp and 40–220 bp were selected to predict the number and distributions of *Msp*I-digested fragments. The gene locations were downloaded from the Ensembl Genome Browser (http://www.ensembl.org/Sus_scrofa/Info/Index). The upstream regions were 5 kb upstream regions to the transcription start sites, and the downstream regions were 5 kb downstream regions to the transcription end sites. Additionally, 5′ untranslated (5’UTR), coding sequence (CDS), intronic and 3′ untranslated (3’UTR) and non-coding regions were denoted the same to the Ensembl Genome Browser. The outside regions of the upstream, 5’UTR, CDS, intron, 3’UTR, downstream, and non-coding regions were defined as the intergenic regions. CGI locations were downloaded from UCSC (http://hgdownload.soe.ucsc.edu/goldenPath/susScr3/database/). The 2 kb upstream and downstream regions of the CGIs were defined as the CGI shores. The 2 kb upstream and downstream regions of the CGI shores were defined as the CGI shelves. The outside regions of the CGIs, shores and shelves were defined as the inter CGI regions. The locations of repetitive elements were also downloaded from UCSC (http://hgdownload.soe.ucsc.edu/goldenPath/susScr3/database/). The simulations and calculations were completed by Perl and R scripts.

### RRBS Library Preparation and Sequencing

The library constructions and sequencing services were provided by RiboBio Co., Ltd. (Guangzhou, China). The processes and procedures for building RRBS libraries were based on the technical processes described by previously published RRBS studies [7, 20]. Briefly, the porcine ovarian genomic DNA was first extracted using a DNeasy Blood & Tissue Kit (Qiagen, Beijing). After checking the quality of the extracted DNA, the ovarian genomic DNA was digested overnight with *Msp*I (New England Biolabs, USA). The sticky ends were filled with CG nucleotides and 3′ A overhangs were added to the *Msp*I-digested segments. Second, the methylated Illumina sequencing adapters with 3′ T overhangs were ligated to the digested segments; then, the products were purified. Afterwards, the 40–110 bp, 110–220 bp and 40–220 bp fragments were separately selected and converted by bisulfite using an EZ DNA Methylation Gold Kit (Zymo Research, USA). Finally, libraries of 40–110 bp, 110–220 bp and 40–220 bp fragments were PCR amplified, and each library was sequenced with one lane of an Illumina HiSeq 2500 as well as 100-bp paired-end reads (PE100). All reads were trimmed using Trim Galore (v0.4.0) software (Babraham Bioinformatics, http://www.bioinformatics.babraham.ac.uk/projects/trim_galore/) and a Phred quality score of 20 as the minimum. The adaptor pollution reads and multiple N reads (where *N* > 10% of one read) were removed off to generate the clean reads. The quality control checks were performed by FastQC (v0.11.3) software (Babraham Bioinformatics, http://www.bioinformatics.babraham.ac.uk/projects/fastqc/). The clean RRBS data were mapped to the porcine reference genome (Sscrofa 10.2, http://www.ensembl.org/Sus_scrofa/Info/Index) and were called the DNA methylation by Bismark software (v0.14.5) [23]. The first two nucleotides were trimmed from all the second read sequences to blunt-end the *Msp*I site. For the overlapped reads, only the methylation calls of read 1 were used for in the process by Bismark with the option “-- no_overlap”, in order to avoid scoring the overlapping methylation calls twice. The bisulfite conversion rates were calculated as the number of covered cytosines in the non-CpG context, which were converted, was divided by the total number of covered cytosines in the non-CpG context [20]. The conversion efficiencies of the fragment sizes of 40–110 bp, 110–220 bp and 40–220 bp were 99.22, 99.60 and 99.31%, respectively. The RRBS data of these three fragment sizes were submitted to the European Nucleotide Archive (accession number: PRJEB14111).

### Sub-sampling of RRBS Data and Bioinformatic Analysis

To assess the cost performances of different fragment sizes with different dataset sizes, we randomly sampled subsets of the paired reads from the whole RRBS data of 40–110 bp, 110–220 bp and 40–220 bp in triplicate and then investigated the features of these sub-sampled data. We respectively triplicated the generations of 10, 7, and 15 increasingly sub-sampled data sets for the fragment sizes of 40–110 bp, 110–220 bp and 40–220 bp. During the sampling process, the minimum number of reads was set as 10 million, which were paired reads (5 million from read 1 and 5 million from read 2). Then the dataset size was increasingly paired by 10 million reads (5 million from read 1 and 5 million from read 2). All sub-sampled data were aligned to the porcine genome, and the detected CpG sites were extracted by Bismark [23]. The mapping efficiencies for the different sub-samples of data were the same as those of the total data, suggesting that the sampling process was successful. Uniquely mapped reads were retained for further calculations. For RRBS data, the detected CpG sites with more than three covered reads were remained for further analyses. The average values of triplications were presented in this study.

## Results

### Length Distribution of *Msp*I-digested Segments in Three Fragment Sizes

Based on the simulating processes of one previous study [24], the porcine reference genome (Sscrofa10.2) was digested by *Msp*I in the simulation. Fragment sizes of 40–110 bp, 110–220 bp and 40–220 bp were selected to evaluate the performance of RRBS on pigs. The fragment sizes of 40–110 bp, 110–220 bp and 40–220 bp contained 385,352, 281,798 and 664,080 *Msp*I-digested segments (Table 1) and covered 3,550,514, 3,718,483 and 7,234,567 CpG sites, respectively (Table 1). The average counts of CpG sites per segment was 9.21, 13.20 and 10.89 in the fragment sizes of 40–110 bp, 110–220 bp and 40–220 bp (Table 1), respectively.

**Table 1:** The contents of the three fragment sizes

Fragment Size	Number of CpG Sites	Number of Segments
40–110 bp	3,550,514	385,352
110–220 bp	3,718,483	281,798
40–220 bp	7,234,567	664,080

**Fig. 1:**
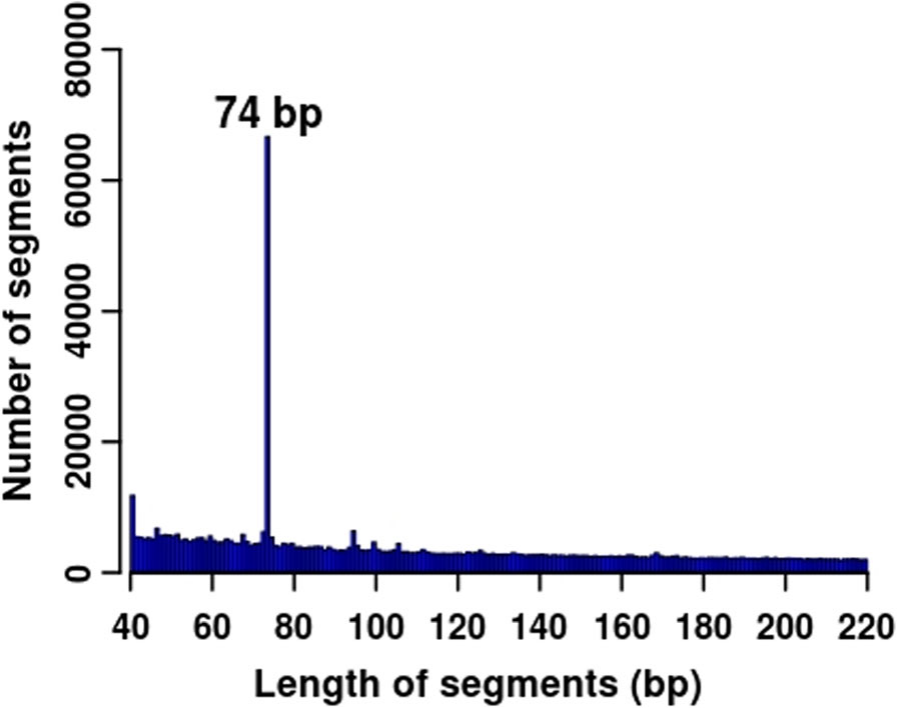
The length distribution of the *Msp*I-digested segments between 40 and 220 bp

The length distribution of the *Msp*I-digested segments between 40 and 220 bp is shown in Fig. 1. Compared with that of 110–220 bp, the specific feature of the 40–110 bp fragment size was that it harbored a high distribution peak at 74 bp, which contained 66,733 *Msp*I-digested segments (Fig. 1). Moreover, we found that 95.40% of 74-bp length digested segments overlapped with the repetitive elements. Then, the single base sequences belonging to the 74-bp length digested segments were extracted, and these sequences were aligned with the porcine reference genome by bowtie2 (v2.2.5) [25]. We found that there were only 10.61% uniquely aligned segments; moreover, 89.39% of the 74-bp length digested segments had multiple alignments.

### Mapping Efficiencies of These Three Fragment Sizes

**Fig. 2:**
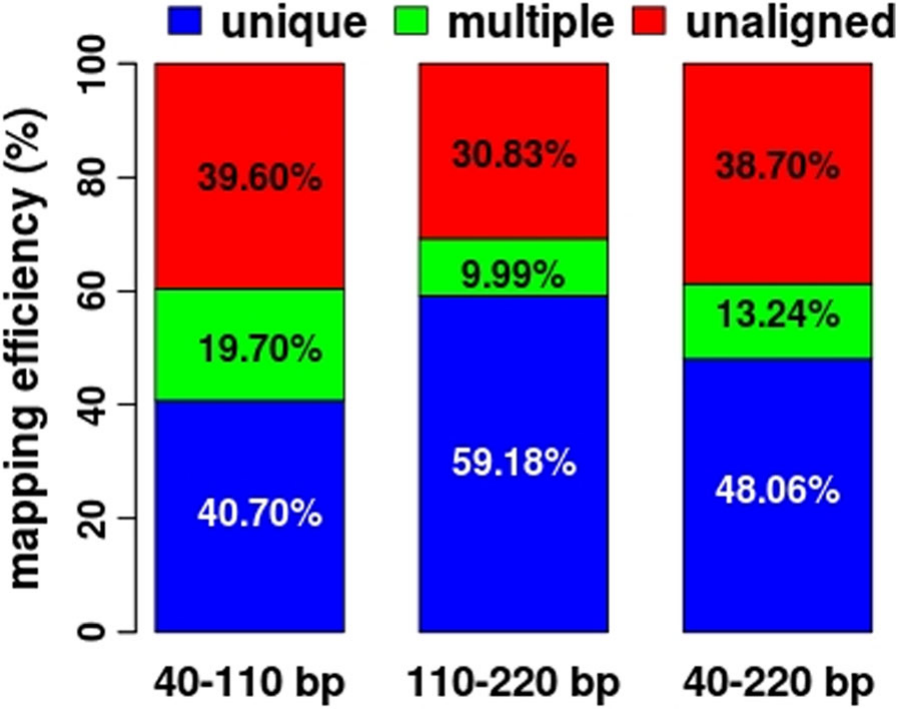
Mapping efficiencies of the 40–110 bp, 110–220 bp and 40–220 bp fragment sizes

To characterize the RRBS performances of these three fragment sizes, we generated more than 100, 70 and 150 million RRBS reads for the 40–110 bp, 110–220 bp and 40–220 bp libraries from the same DNA sample, respectively. Bismark [23] was used to map these RRBS data to the porcine genome. We found that the unique mapping efficiencies were 40.70, 59.18 and 48.06% for the 40–110 bp, 110–220 bp and 40–220 bp libraries, respectively. The multiple mapping efficiencies were 19.70, 9.99 and 13.24% for the 40–110 bp, 110–220 bp and 40–220 bp libraries, respectively (Fig. 2). These results indicated that the fragment size of 110–220 bp had the highest unique mapping alignment with the lowest multiple alignment (Fig. 2). Moreover, compared with the mapping efficiency of 110–220 bp, the relatively higher multiple alignments and the relatively lower unique mapping alignments of 40–110 bp and 40–220 bp libraries suggested that the 74-bp length digested segments, which were highly overlapped with the repetitive elements, might reduce the unique mapping efficiency.

### Optimal Sequencing Quantities for These Three Fragment Sizes

To investigate the optimal sequencing quantities of these fragment sizes, we randomly sampled different subsets of reads from the whole RRBS data of these three fragment sizes and generated 10, 7, and 15 increasingly sub-sampled data sets for the fragment sizes of 40–110 bp, 110–220 bp and 40–220 bp in triplicate, respectively. Uniquely mapped reads and the detected CpG sites with ≥3 covered reads (3×) were retained for further calculations.

**Fig. 3:**
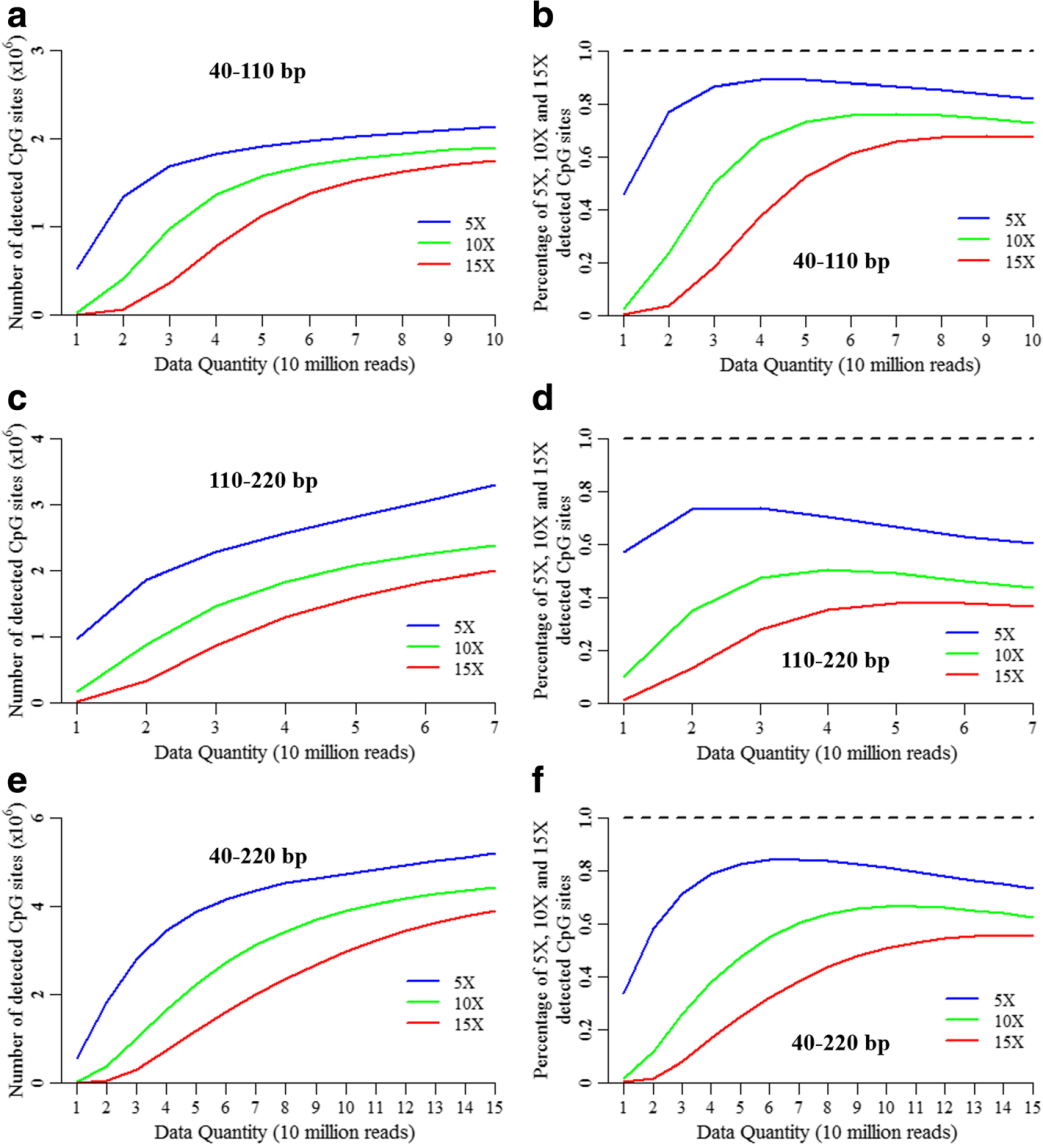
Distributions of detected CpG sites in the differently sub-sampled RRBS data. The number distributions of detected CpG sites with ≥5, 10 and 15 covered reads (5X, 10X and 15X) in the differently sub-sampled RRBS data for 40–110 bp (**a**), 110–220 bp (**c**) and 40–220 bp (**e**) fragment sizes in triplications. The percentages of 5X, 10X and 15X detected CpG sites over the 3× detected CpG sites in the differently sub-sampled RRBS data for 40–110 bp (**b**), 110–220 bp (**d**) and 40–220 bp (**f**) fragment sizes in triplications

The number distributions and sequencing saturations of the detected CpG sites with ≥5, 10 and 15 covered reads (5X, 10X and 15X) are shown in Fig. 3. As expected, the numbers of 5X, 10X and 15X detected CpG sites increased with increasing data sizes for these three fragment sizes (Fig. 3a–e). However, the increasing speed of 10X detected CpG sites decreased when the data size was more than 70, 50 and 110 million reads for the 40–110 bp, 110–220 bp and 40–220 bp fragment sizes, respectively (Fig. 3a–e). Moreover, the saturations and percentages of the 10X detected CpG sites over the 3× detected CpG sites also descended when the data size was more than 70, 50 and 110 million reads for the 40–110 bp, 110–220 bp and 40–220 bp fragment sizes, respectively (Fig. 3d–f), suggesting that the cost effectiveness of these fragment sizes might decrease with a higher dataset size.

### Sequencing Depth of These Three Fragment Sizes

**Fig. 4:**
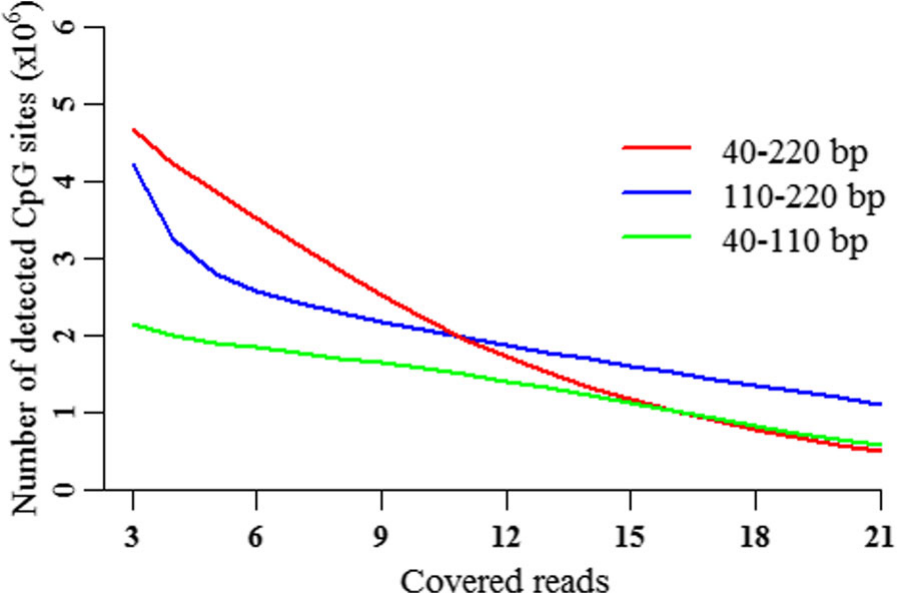
Distributions of detected CpG sites versus the differently covered depth for the three fragment sizes in 50 million reads in triplications

Considering the cost and acquired number of detected CpG sites, we selected the data size of 50 million reads to evaluate the sequencing depth of these three fragment sizes. Given a 50-million read dataset size, the fragment size of 40–220 bp detected more CpG sites with 5X than 40–110 bp and 110–220 bp (Fig. 4). The 40–220 bp fragment size detected almost the same number of CpG sites with 10X as for 110–220 bp and detected more sites than for 40–110 bp. However, the 40–220 bp fragment size detected fewer CpG sites with 15X than for 110–220 bp, while it was almost the same as that for 40–110 bp (Fig. 4). These results suggested that the average sequencing depth of the detected CpG sites in the 110–220 bp fragment size appeared to be deeper than in the 40–110 bp and 40–220 bp fragment sizes.

### Distribution of Detected CpG Sites in These Three Fragment Sizes

**Fig. 5:**
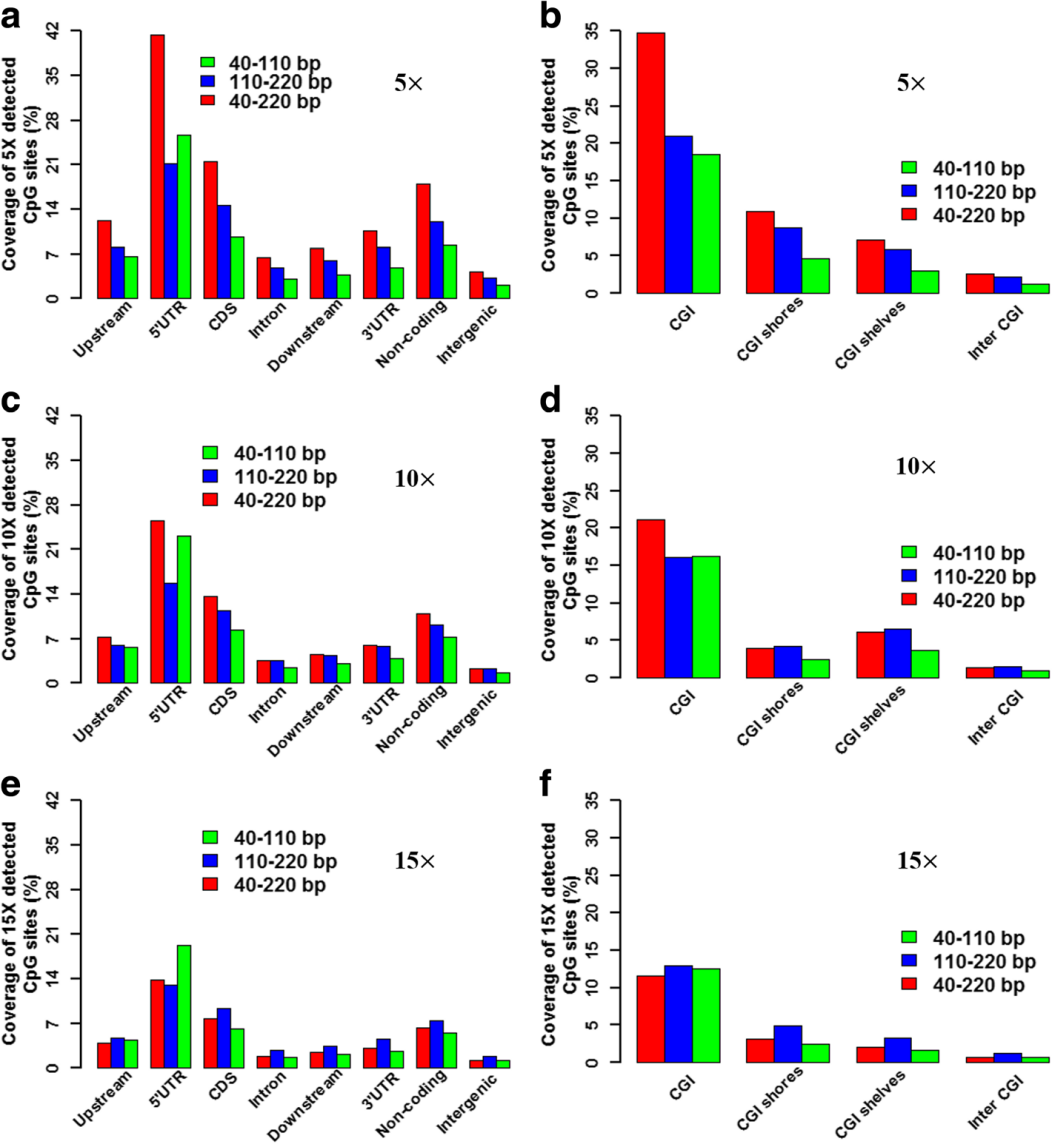
Coverage of the detected CpG sites of these three fragment sizes across the gene-related and CGI-related regions for the whole porcine genome. The coverages of 5X, 10X and 15X detected CpG sites across the gene-related regions for 40–110 bp (**a**), 110–220 bp (**c**) and 40–220 bp (**e**) fragment sizes in triplications. The coverages of 5X, 10X and 15X detected CpG sites across the CGI-related regions for 40–110 bp (**b**), 110–220 bp (**d**) and 40–220 bp (**f**) fragment sizes in triplications

Given a 50-million dataset size, the fragment size of 40–220 bp detected the highest number of CpG sites with 5X within gene- and CGI-related regions compared with 40–110 bp and 110–220 bp fragment sizes (Fig. 5a, b). For the CpG sites with 10X, the 40–220 bp fragment size detected the highest number of CpG sites within gene-related regions and CGIs (Fig. 5c, d), but the 110–220 bp fragment size detected the highest number of CpG sites within the CGI shores and CGI shelves compared with the other two fragment sizes (Fig. 5d). Furthermore, considering the CpG sites with 15X, the fragment size of 110–220 bp detected the most CpG sites within gene- and CGI-related regions compared with the 40–110 bp and 40–220 bp fragment sizes (Fig. 5e, f). Interestingly, considering the CpG sites with 5X, 10X and 15X, the 40–110 bp fragment size always detected more CpG sites within the 5’UTR regions than those with a fragment size of 110–220 bp (Fig. 5a–e).

## Discussion

DNA methylation is an important epigenetic modification that plays a critical function in many biological processes. Profiling of the genome-wide DNA methylation allows for investigations of DNA methylation dynamics and epigenetic mechanisms of many key biological processes [10, 26]. Compared with the other sequencing strategies, RRBS is a cost-effective pipeline to generate genome-wide DNA methylation at the single-nucleotide resolution [8, 20]. By enriching in the CpG-rich regions and relying on bisulfite sequencing methods, RRBS reduces the sequencing requirement and enhances the sequencing depth and accuracy of DNA methylation information for the targeted regions of the genome-scale DNA methylation [27]. In this study, the 40–110 bp, 110–220 bp and 40–220 bp fragment sizes were selected to assess the capabilities, data utilization efficiencies and cost performances of RRBS in pigs. We found that there was a length distribution peak at 74 bp, which highly overlapped with the repetitive elements and might reduce the unique mapping alignment. The 110–220 bp library displayed the highest unique mapping alignment and the lowest multiple alignment. When the data sizes are more than 70, 50 and 110 million reads for the fragment sizes of 40–110 bp, 110–220 bp and 40–220 bp, respectively, the cost effectiveness of these fragment sizes might decrease. Given a 50-million dataset size, the average sequencing depth of the detected CpG sites in the 110–220 bp fragment size appeared to be deeper than in the 40–110 bp and 40–220 bp fragment sizes.

In vertebrate genomes, the 40–220 bp fragment size is commonly used to resolve the mammalian methylome with RRBS. With a fragment size of 40–220 bp, the alignment efficiencies were approximately 30–40% for different mouse cells by 36-bp single-end sequencing [6]. However, the percentages of uniquely mapped reads only ranged from 27.0 to 32.7% for zebrafish with 100-bp single-end sequencing [11]. The mapping ratio was approximately 40–50% for human embryos and sperm cells with PE100 [28]. The uniquely mapped reads were approximately 48% for pigs with 50-bp paired-end sequencing (PE50) [10]. In this study, we also found that the unique mapping efficiency was approximately 48% for the 40–220 bp fragment size in pigs (Fig. 2). However, the unique mapping efficiency increased to approximately 60% for the 110–220 bp fragment size in pigs (Fig. 2).

Multiple factors contributed to the relatively low utilization efficiency for the 40–220 bp fragment size. First, there were many short segments in the range of 40–220 bp (Fig. 1). The short segments were easily aligned to multiple locations with the present mapping model, which reduced the unique mapping ratio. Second, the selection of the sequencing strategy might be not appropriate. For example, considering the case of sequencing the 40–220 bp library with PE50, there was always a region (i.e., 50 bp for 150-bp segments and 120 bp for 220-bp segments in the center) that could not be covered by any read, resulting in loss of methylation information harbored by the uncovered regions. Third, large repetitive sequences might be located in the 40–220 bp fragment size, aggravating the ratio of multiple alignments [29].

One previous study suggested that short reads and a large number of repetitive elements might decrease the unique mapping efficiency of RRBS data [29]. In this study, we found that 95.40% of the 74-bp *Msp*I-digested segments overlapped with repetitive elements, and 89.39% of these segments were aligned to multiple locations. Furthermore, the unique mapping efficiency of the 40–110 bp fragment size was 40.10% (Fig. 2), which was the lowest compared with the other two fragment sizes (Fig. 2). In addition, one previous study recommended that there are redundant microsatellites, one of the repetitive elements, located in the *Msp*I-digested segments for 40–220 bp in the mouse genome [30], and these microsatellites might result in the low alignment efficiencies of the RRBS data from mouse cells by the 36-bp single-end sequencing [6]. These results showed that repetitive elements might decrease the unique mapping efficiency.

Given the 50, 70, 90 and 110 million reads of RRBS data, the 40–220 bp fragment size detected 2,233,833; 3,122,473.33; 3,699,966 and 4,058,334.33 CpG sites with 10X, respectively (Fig. 3e). Given the 130 and 150 million reads of RRBS data, the 40–220 bp fragment size detected 4,285,318.67 and 4,438,622.67 CpG sites with 10X, respectively (Fig. 3e), respectively. Compared with the 50 million reads, the dataset sizes of 70, 90 and 110 million reads increased by 40, 80 and 120%, and the numbers of detected CpG sites with 10X increased by 39.78, 65.63 and 81.68%, respectively (Fig. 3e). However, compared with the 50 million reads, the dataset sizes of the 130 and 150 million reads increased by 160 and 200%, but the numbers of detected CpG sites with 10X only increased by 91.84 and 98.70%, respectively (Fig. 3e). As a result, when the data set was more than 110 million reads, the cost-efficiency decreased for the fragment size of 40–220 bp.

The selections of fragment sizes could affect the numbers and sequencing depth of detected CpG sites as well as the mapping efficiency. For the 50 million reads, the fragment size of 40–220 bp detected 2,233,833 CpG sites with 10X, which was almost the same number of CpG sites with 10X for 110–220 bp, while it was more than for 40–110 bp (Fig. 4). However, the average sequencing depth per detected CpG site was 18.82 for the fragment size of 40–220 bp, which was lower than for 110–220 bp (26.56) and 40–110 bp (21.60). Moreover, the unique mapping efficiency of 40–110 bp fragment size was 40.70% with PE100, but it approximately increased to 60% for the 110–220 bp fragment size (Fig. 2). The unique mapping efficiency of 50–150 bp fragment size was 38.3% with PE100 in sheep, but it increased to 61.4% for the 150–220 bp fragment size [5]. Therefore, the selections of fragment sizes could affect the numbers and sequencing depth of detected CpG sites as well as the cost-efficiency. Taken together, when designing methylome studies using RRBS, researchers should consider the fragment size, mapping efficiency, sequencing depth, covered CpG sites, and dataset size. No single solution of RRBS is optimal in all circumstances for investigating genome-scale DNA methylation.

## Conclusions

In this study, our results demonstrated that selections of fragment sizes could affect the numbers and sequencing depth of detected CpG sites as well as the cost-efficiency. No single solution of RRBS is optimal in all circumstances for investigating genome-scale DNA methylation. This work provides the useful knowledge on designing and executing RRBS for investigating the genome-wide DNA methylation in tissues from pigs.

## Abbreviations

10X: ≥ 10 covered reads
15X: ≥ 15 covered reads.
3’UTR: 3′untranslated region
3X: ≥ 3 covered reads
5’UTR: 5′Untranslated region
5X: ≥ 5 covered reads
CDS: Coding sequence
CGIs: CpG islands
PE100: 100-bp paired-end reads
PE50: 50-bp paired-end sequencing
RRBS: Reduced representation bisulfite sequencing
WGBS: Whole-genome bisulfite sequencing
